# Use of Nasotracheal Intubation during General Anesthesia in Two Ponies with Tracheal Collapse

**DOI:** 10.3389/fvets.2018.00042

**Published:** 2018-03-13

**Authors:** Keila K. Ida, Aurélie Sauvage, Alexandra Gougnard, Magda Grauwels, Didier Serteyn, Charlotte Sandersen

**Affiliations:** ^1^Anesthésiologie et Réanimation Vétérinaires, Département Clinique des Animaux de Compagnie et des Équidés, Faculté de Médecine Vétérinaire, Université de Liège, Liège, Belgium; ^2^Chirurgie et Clinique Chirurgicale des Petits Animaux, Département Clinique des Animaux de Compagnie et des Équidés, Faculté de Médecine Vétérinaire, Université de Liège, Liège, Belgium

**Keywords:** horses, risk factors, intubation, emergencies, recovery from anesthesia

## Abstract

Ponies with tracheal collapse may have an increased anesthetic risk due to airway obstruction during induction and recovery. To our knowledge, there are no anesthetic descriptions of these patients, despite a reported 5.6% incidence and 77% mortality rate. Two Shetland ponies with tracheal collapse, a 12-year-old male (pony 1) and a 27-year-old female (pony 2), were referred for right eye enucleation due to a perforating corneal ulcer and severe recurrent uveitis, respectively. Pony 1 was stressed, had lung stridor and hyperthermia, and developed inspiratory dyspnea with handling. Radiography confirmed collapse of the entire trachea as well as inflammation of the lower airways. Corticosteroids and bronchodilators were administered by nebulization for 1 week before surgery. Pony 2 had a grade III/VI mitral murmur and a clinical history of esophageal obstructions and tracheal collapse requiring tracheostomy. Both ponies were premedicated with acepromazine and xylazine; anesthesia was induced with midazolam and ketamine. Nasotracheal intubation was performed in left lateral recumbency with extension of the neck and head and was guided by capnography. The nasotracheal tube consisted of two endotracheal tubes attached end-to-end to create a tube of adequate length and diameter. Pony 2 was orotracheally intubated during surgery and later reintubated with a nasotracheal tube. Anesthesia was maintained with isoflurane using volume-controlled ventilation. Analgesia was provided by a retrobulbar blockade with mepivacaine and lidocaine. Cardiovascular support consisted of lactated Ringer’s solution and dobutamine. After surgery, the ponies were administered xylazine and supplemented with oxygen through the nasotracheal tube. Recovery was assisted by manual support of the head and tail. Successful extubation was achieved following butorphanol administration after approximately 1 h in standing position. Both ponies were discharged from the clinic a few days after surgery.

## Introduction

### Case Presentations

Pony 1 was a 12-year-old male Shetland pony weighing 84 kg with a perforating corneal ulcer of the right eye that was referred for enucleation. The clinical history included tracheal collapse, inflammation of the lower respiratory airways, and laminitis. On physical examination, the pony was stressed, dyspneic, and hyperthermic, and had nasal discharge and crackles on lung auscultation. Radiographic examination confirmed collapse of the entire trachea and showed a bronchoalveolar pattern in the lungs. Surgery for eye removal was scheduled after 1 week of nebulization with corticosteroids and bronchodilators, and treatment with oral suxibuzone. At the time of surgery, the pony was no longer hyperthermic, and had a hematocrit of 44% and a total plasma protein concentration of 53 g/l.

Pony 2 was a 27-year-old female Shetland pony weighing 136 kg with severe recurrent uveitis in the right eye that was referred for enucleation. The left eye had been enucleated 4 months prior in our institution. At that time, severe tracheal collapse requiring tracheostomy occurred 6 h after recovery from anesthesia. The animal had a grade III/VI mitral valve murmur and was blind in the right eye. At presentation, the pony was calm, vital parameters were within normal limits, and no clinical signs of tracheal collapse were observed. The pony had a hematocrit of 32% and a total plasma protein concentration of 67 g/l.

### Anesthesia and Airway Management

**Figure 1:**
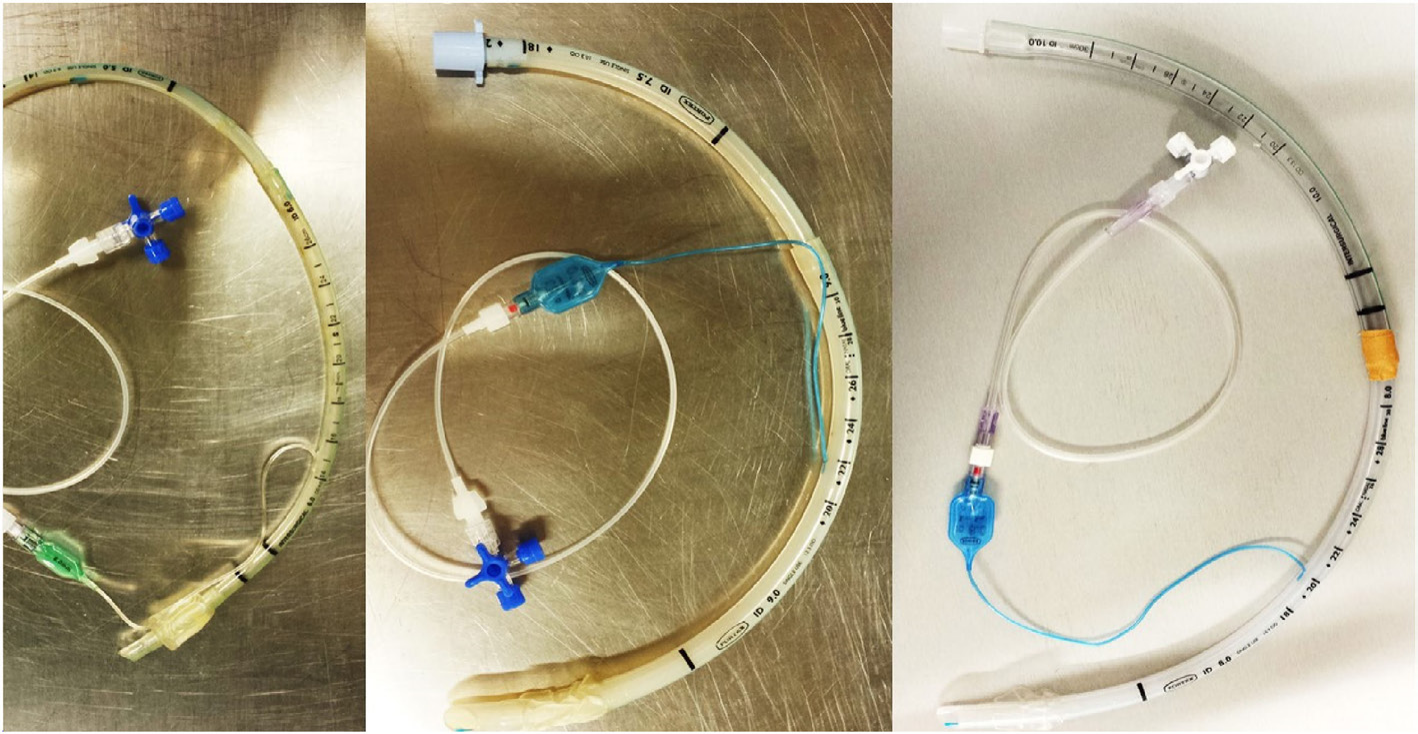
Three pairs of endotracheal tubes connected end to end were prepared on the day before surgery: tubes of 5.0-mm internal diameter (ID) with 6.0-mm ID, 7.5-mm ID with 9.0-mm ID, and 8.0-mm ID with 10-mm ID. The proximal connector of the more distal tube of each pair was removed and attached with adhesive tape to the distal extremity of the more proximal tube. The balloon cuff of the more proximal tube was removed, while the balloon cuff of the more distal tube was connected to a tubing extension and a three-way stopcock that was maintained outside the animal’s mouth. It could have been used to pull out the distal tube in case of detachment.

Three endotracheal tubes were prepared on the day before surgery, each consisting of a pair of endotracheal tubes connected end-to-end. Two pairs of endotracheal tubes [5.0-mm internal diameter (ID) and 6.0-ID; and 7.5-ID and 9.0-ID] were attached to each other with the largest tube positioned distally. One pair of endotracheal tubes (8.0-ID and 10-ID) was attached with the smallest tube positioned distally. The proximal connector of the more distal tube of each pair was removed and attached with adhesive tape to the distal extremity of the more proximal tube. The balloon cuff of the more proximal tube was removed, while the balloon cuff of the more distal tube was connected to a tubing extension and a three-way stopcock (Figure 1).

Similar anesthetic protocol and airway management were used for both ponies on different days. Materials for a tracheostomy were prepared in case of emergency. Food was withdrawn for 12 h with free access to water prior to anesthesia. On the day of surgery (and for 5 days following surgery), the animals were administered gentamicin (6.6 mg/kg IV, SID), sodium penicillin (22,000 mg/kg IM, SID), and flunixin meglumine (1.1 mg/kg IV; SID). Acepromazine (0.1 mg/kg) was administered intramuscularly and, after 1 h, xylazine (0.6 mg/kg) was administered through a 14-G catheter fixed in the right jugular vein. In pony 2, butorphanol (0.02 mg/kg IV) was administered with acepromazine and xylazine as premedication. Anesthesia was induced with midazolam (0.06 mg/kg IV) and ketamine (2.2 mg/kg IV).

Once in left lateral recumbency, the ponies were moved to the surgical theater on a surgical table. Lidocaine (10%) was sprayed into the right nostril and the head was positioned in full extension to create an angle of 180° between the neck and the mandible. In pony 1, the 6.0-5.0-ID pair of endotracheal tubes with silicone spray applied externally was introduced into the right nostril and forwarded to the trachea, guided by capnography. Inflation of the cuff indicated that the endotracheal tubes were too small. Intubation with an 11-ID endotracheal tube was not successful and it was replaced by the 8.0-10-ID pair of endotracheal tubes. Nasotracheal intubation was accomplished 11 min after induction of anesthesia, during which time the pony was breathing spontaneously, with oxygen supplementation through the left nostril. In pony 2, nasotracheal intubation was performed similarly and was accomplished within 2 min after induction of anesthesia, using the 7.5-9.0-ID pair of endotracheal tubes. The tubes were attached to the nostrils with adhesive tape. The balloon cuff was kept inside the mouth of the animal, the tubing extension was kept partially within the mouth, and the remaining tubing extension and three-way stopcock were maintained outside the mouth. None of the three pieces was in contact with the tracheal wall.

The animals were mechanically ventilated (Tafonius, Vetronics, Devon, UK) using volume-controlled ventilation with a respiratory rate (RR) of 14–16 bpm, tidal volumes (*V*_T_) of 8–13 and 8–11 ml/kg for ponies 1 and 2, respectively, and peak inspiratory pressure of 20–25 cmH_2_O, which allowed for an end-tidal carbon dioxide concentration (ETCO_2_) of 35–50 mmHg. After a few minutes of mechanical ventilation of pony 2, the absence of capnography curves while breathing movements were present indicated accidental extubation. The endotracheal tube was removed and replaced by a 16-ID tube introduced orotracheally; this tube was left in place throughout the surgery.

During anesthesia, the expired fraction of isoflurane was maintained at 0.8–1.3%. Heart rate and rhythm were monitored using an electrocardiogram (Tafonius). A 20-G catheter was inserted into the right metatarsal artery for monitoring invasive arterial pressure and for acquiring samples for blood gas analysis (Tables 1 and 2). Lactated Ringer’s solution (10 ml/kg/h) and dobutamine (0.5–1.0 µg/kg/min) were administered for maintaining the mean arterial pressure at 70–85 mmHg. Results of blood gas analysis (Cobas b 123, Roche, Brussels, Belgium) were within reference values for the species (Tables 1 and 2).

**Table 1:** Oxygenation, arterial blood gas parameters, and plasma electrolyte concentrations of a 12-year-old male pony under isoflurane anesthesia with volume-controlled ventilation through a nasotracheal tube.

Analyte (unit)	Minutes after induction of anesthesia	
	
	90	120
FIO_2_	0.82	0.85
pH	7.386	7.363
PaCO_2_ (mmHg)	47.4	51.7
PaO_2_ (mmHg)	422.0	442.3
SaO_2_ (%)	98.4	99.1
ETCO_2_ (mmHg)	42	42
Vd/*V*_T_ (%)	11.4	18.8
PAO_2_ (mmHg)	534	550
P(A-a)O_2_ (mmHg)	112	108
$HCO_{3^{-}}$ (mmol/l)	27.8	28.7
BE (mmol/l)	2.46	2.68
Hematocrit (%)	35.8	36.3
Na^+^ (mmol/l)	120.7	123.8
K^+^ (mmol/l)	2.89	2.84
Cl^−^ (mmol/l)	94.1	93.7
Ca^2+^ (mmol/l)	1.164	1.242

*FIO_2_, fraction of inspired oxygen; PaCO_2_, partial pressure of carbon dioxide in arterial blood; PaO_2_, partial pressure of oxygen in arterial blood; SaO_2_, oxygen saturation; ETCO_2_, end-tidal carbon dioxide concentration; Vd/*V*_T_, alveolar dead space-to-tidal volume ratio; PAO_2_, partial pressure of alveolar oxygen; P(A-a)O_2_, alveolar-arterial oxygen pressure gradient; $HCO_{3^{-}}$, bicarbonate concentration; BE, base excess*.

**Table 2:** Oxygenation, arterial blood gas parameters, and plasma electrolyte concentrations of a 27-year-old female pony under isoflurane anesthesia with volume-controlled ventilation through an orotracheal tube.

Analyte (unit)	40 min after induction of anesthesia
FIO_2_	0.96
pH	7.411
PaCO_2_ (mmHg)	39.9
PaO_2_ (mmHg)	527.9
SaO_2_ (%)	98.8
ETCO_2_ (mmHg)	30
Vd/*V*_T_ (%)	24.8
PAO_2_ (mmHg)	644
P(A-a)O_2_ (mmHg)	116
$HCO_{3^{-}}$ (mmol/l)	24.8
BE (mmol/l)	0.16
Hematocrit (%)	26.3
Na^+^ (mmol/l)	134.2
K^+^ (mmol/l)	3.47
Cl^−^ (mmol/l)	105.8
Ca^2+^ (mmol/l)	1.343

*FIO_2_, fraction of inspired oxygen; PaCO_2_, partial pressure of carbon dioxide in the arterial blood; PaO_2_, partial pressure of oxygen in the arterial blood; SaO_2_, oxygen saturation; ETCO_2_, end-tidal carbon dioxide concentration; Vd/*V*_T_, alveolar dead space-to-tidal volume ratio; PAO_2_, partial pressure of alveolar oxygen; P(A-a)O_2_, alveolar-arterial oxygen pressure gradient; $HCO_{3^{-}}$, bicarbonate concentration; BE, base excess*.

The partial pressure of alveolar oxygen (PAO_2_) was calculated according to a standard formula: PAO_2_ = [FIO_2_ × (Patm − PH_2_O)] − (PaCO_2_/0.8), where FIO_2_ is the fraction of inspired oxygen; Patm is the atmospheric pressure at the time of anesthesia (770 mmHg in Liège, Belgium per http://www.worldweatheronline.com); PH_2_O is the partial pressure of water vapor (defined as 47 mmHg, at a rectal temperature of 37°C); PaCO_2_ is the partial pressure of carbon dioxide in arterial blood; and 0.8 is the respiratory quotient; The alveolar dead space-to-tidal volume ratio (Vd/*V*_T_) was calculated as: Vd/*V*_T_ = [(PaCO_2_ − ETCO_2_)/PaCO_2_] × 100.

Surgery was started following a retrobulbar blockade with 2.5 ml lidocaine (2%) and 2.5 ml mepivacaine (1%) and using a 20G × 70 mm needle that was inserted caudal to the orbital rim through the periorbital fascia. The procedure lasted 125 and 45 min in ponies 1 and 2, respectively. At the end of surgery, isoflurane was interrupted and RR was decreased to 1 bpm for recovering spontaneous breathing. In pony 2, the orotracheal tube was removed and replaced by a nasotracheal tube (7.5–9.0 ID) that was introduced with capnography guidance and attached to the nostrils with adhesive tape.

The animals were moved to the recovery room where they were maintained in left recumbency and supplemented with oxygen (6 l/min) through the nasotracheal tube. Xylazine (0.2 mg/kg IV) was administered, and the animal was allowed to recover with manual support of the tail and head by two veterinarians. Sternal recumbency and a standing position were accomplished in the first attempt at 20 and 24 min after disconnection from the ventilator in pony 1, and at 25 and 35 min in pony 2. Both ponies were mildly ataxic but recovery was overall calm. They were walked to their boxes and monitored until full recovery.

After an hour in standing position, the first attempt to extubate pony 1 was unsuccessful due to severe tachypnea and cough; therefore, the tube was not removed. Successful extubation without tachypnea and cough was achieved 5 min following administration of butorphanol (0.02 mg/kg IV) at 1h15 and 1h35 after achieving a standing position in ponies 1 and 2, respectively. No episode of respiratory distress was observed for 5 and 7 days, respectively, and the ponies were discharged from the clinic.

## Background

In ponies, non-congenital tracheal collapse occurs as a progressive degeneration of the hyaline cartilage rings and weakening of the dorsal trachealis muscle. It is a relatively common disease associated with a poor prognosis in this breed (1). In an American university teaching hospital, tracheal collapse was observed in 13 of 231 (5.6%) American Miniature Horses examined in a 22-year period; mortality rate was 77% (1). In our institution, during a 4-year period, tracheal collapse was observed in 10 of 473 ponies (2.1%) and mortality rate was 40%.

Clinical signs of tracheal collapse include inspiratory stridor, cough, and dyspnea, which can lead to airway obstruction, cyanosis, and death (2). Medical treatment consists of anti-inflammatory drugs to treat concurrent respiratory tract disease and, in mild cases, keeping the animal calm to avoid respiratory distress (3). Surgical treatment may be indicated in severe cases, but the procedure is rare, with only four ponies described in the literature (2–4).

Although tracheal collapse is a life-threatening disease, the owners often are not aware of its presence, since respiratory distress may occur only during stressful conditions such as anxiety, pain, hypothermia, and hyperthermia (3). These conditions can be observed during recovery from anesthesia and, therefore, ponies with tracheal collapse may have increased anesthetic risk, especially if the diagnosis is not known at the time of the procedure. The exact risk is unknown since in previous reports the tracheal collapse had been surgically corrected and therefore, was not an issue during recovery from anesthesia (2–4). Reports describing anesthesia or airway management of ponies with tracheal collapse undergoing surgical procedures other than insertion of tracheal stents were not found. In our institution, 5 of 10 ponies with tracheal collapse required general anesthesia for colic surgery or enucleation, including the 2 described here. Two other ponies were euthanized during surgery; however, 1 pony, together with pony 2 during its first enucleation surgery, had undiagnosed tracheal collapse and developed respiratory complications at recovery. These data suggest that ponies with tracheal collapse are at a higher anesthetic risk for respiratory complications and that this risk can be decreased by carefully planning anesthetic and airway management prior to surgery. Therefore, the goal of this case report was to describe the anesthesia and airway management of two ponies with tracheal collapse, providing guidelines that can be used in similar cases to decrease anesthetic risk.

## Discussion

The anesthetic protocol described here was aimed at preventing any stressful condition, such as anxiety, pain, dysphoria, cough, and hypothermia that could trigger tachypnea and tracheal collapse. Airway management focused on providing adequate oxygenation and ventilation during surgery, as well as oxygen supplementation during recovery from anesthesia.

Pain, anxiety, and dysphoria were prevented by using systemic analgesics, a non-steroidal anti-inflammatory drug (NSAID), sedatives, and retrobulbar blockade (regional anesthesia). Stressful conditions were also prevented during recovery from anesthesia, to avoid tachypnea and tracheal collapse. Analgesia was achieved as part of a multimodal approach using drugs with different mechanisms of action for pain relief. Xylazine produces analgesia through central alpha-2 receptors, providing the sedative and myorelaxant effects required at premedication and recovery from anesthesia. Cardiorespiratory depression, a potential dose-dependent side effect, was minimized by using a low dose of xylazine that could be antagonized if necessary (5), especially in cases of cardiac impairment, such as in pony 2. Ketamine is as a non-competitive antagonist of *N*-methyl-d-aspartate receptors, which are associated with the inhibition of nociceptive central hypersensitization and in the decrease of the incidence of opioid tolerance, which is achieved with subanesthetic doses (6). Flunixine meglumine, a NSAID, is the most frequently used analgesic in horses. Its mechanism of action is based on inhibition of cyclooxygenase and, thus, prostaglandin synthesis, which is associated with inflammatory pain (7). Regional anesthesia through retrobulbar blockade with lidocaine also plays an important role in decreasing afferent nociceptive transmission. Additional analgesics, such as opioids, can be used when clinically indicated. In horses, opioids (mainly butorphanol) are effective antitussives and analgesics that enhance sedation caused by other sedatives, such as alpha-2 agonists (7, 8). The use of opioids in ponies with tracheal collapse would have the advantage of increasing sedation while reducing the dose of the alpha-2-agonist required. This multimodal analgesia is also an important means to prevent coughing during intubation. We would like to clarify that the decision not to use opioids in pony 1 prior to surgery was not an oversight, but rather was based on our constant monitoring for any sign of pain and stress. However, neither pony had clinical signs of pain or sympathetic stimulation, even though isoflurane was maintained at a low concentration (23–53% lower than the minimum alveolar concentration for horses). There were no differences or alterations in the cardiovascular and respiratory response to surgery or in the isoflurane requirements between ponies 1 and 2, even though the latter was administered butorphanol prior to surgery. Both animals were calm during recovery from anesthesia and did not show signs of pain, such as tachycardia or reaction to palpation of the surgical wound, suggesting the multimodal analgesia was effective. As mentioned before, we were prepared to administer additional opioids if the ponies had signs of pain and stress. In fact, during the first extubation attempt, pony 1 started to cough, probably as a reflex due to stimulation caused by removal of the endotracheal tube. This stimulus caused stress to the animal, which triggered respiratory distress and did not allow for extubation without tracheal collapse. At this point, butorphanol was administered, which played an important role in facilitating removal of the tube. The butorphanol was effective in controlling the stress and cough reflex due to its sedative and antitussive effects, and also could have provided analgesia in the event that pain was contributing to the stress (8).

Total intravenous anesthesia has been described previously for surgical treatment of tracheal collapse in a pony (3). We could have used this type of anesthesia in pony 2, since surgery lasted less than 1 h, but because we could not predict the length of surgery in pony 1 (it lasted more than 2 h), isoflurane anesthesia was chosen. Isoflurane also has less of a cumulative effect that could have impaired the quality of recovery from anesthesia (9–11). Alternatively, surgery could have been performed in a standing sedated animal, since transpalpebral enucleation in standing healthy horses has been documented (12, 13). However, standing sedation would not have addressed the anesthetic risk appropriately, particularly considering that pony 1 was in poor physical condition and had pulmonary disease, and pony 2 had mitral insufficiency and was geriatric. Sedation does not allow for ventilatory support through intubation and positive pressure ventilation, nor does it allow for more complete monitoring, such as with direct arterial pressure and capnography, to immediately detect potential complications. Spontaneous ventilation increases the risk of tracheal collapse due to the high negative inspiratory pressure (3). High doses of detomidine may be required during standing enucleation to ensure absence of response to surgical stimulation (13), and may induce dyspnea. In addition, deep sedation with alpha-2 agonists is known to be associated with severe cardiorespiratory depression (5), which would not have been tolerated by the ponies of this report due to their compromised clinical status. Therefore, the use of a balanced protocol with general anesthesia was chosen to minimize stress and episodes of dyspnea, to maintain ventilatory patency, and to minimize cardiovascular depression.

The choice of anesthetic drugs should be based on the clinical assessment of each patient; doses should be titrated to effect. Even though pony 2 had a heart murmur, no cardiovascular complications occurred despite using a similar anesthetic management as that used during prior enucleation of the other eye. In addition, the occurrence of any cardiovascular depression or adverse effect (which was not observed) could be immediately detected and promptly treated since the animal was monitored closely using electrocardiography, invasive blood pressure monitoring, capnography, and arterial blood gas analysis.

The drugs and doses used in the two ponies were appropriate for avoiding apnea after induction of anesthesia, allowing time for intubation and for checking ETCO_2_ values for correct placement of the endotracheal tube. If apnea occurs, the tube can be inserted orotracheally to allow ventilation. In addition, endotracheal intubation to the level of the mid-trachea does not prevent potential tracheal collapse and obstruction distally. We were prepared for an emergency tracheostomy if needed, but neither pony required such intervention. Blood gases were not analyzed during recovery to document adequate management of oxygenation, which was a limitation. The ponies recovered quickly from anesthesia and an arterial blood sample was not collected to avoid additional stimulation or stress. Both ponies were administered supplemental oxygen through the endotracheal tube during the surgical procedure and recovery from anesthesia; no clinical signs of hypoxemia (e.g., cyanosis, tachycardia, and tachypnea) were observed.

Nasotracheal intubation is performed blindly in horses and requires small size endotracheal tubes, which may be misplaced into the esophagus. Accurate placement of the tube within the trachea could be confirmed by capnographic monitoring or intubation could be guided by endoscopy, which adds time to the intubation process compared with orotracheal intubation. Furthermore, a nasotracheal tube is usually narrow to be able to fit through the nares, which increases airway resistance during ventilation and may result in nasal hemorrhage. The choice of nasotracheal (vs. orotracheal) intubation allows the tube to be kept in place after full recovery from anesthesia, until the pony is sufficiently calm to be extubated. Alternatively, orotracheal intubation for surgery could be replaced by nasotracheal intubation for the recovery from anesthesia as was done in pony 2. In this case, orotracheal intubation was preferred because it is faster and the animal was in apnea. Because our attempt to insert an 11-ID endotracheal tube was unsuccessful in pony 1, we determined that the size of the tube in place was adequate for the patient and thus did not require changing for recovery. A nasotracheal tube, but not an orotracheal tube, is well tolerated by a conscious horse. The option of leaving the orotracheal tube in place during recovery (14), can be considered if nasotracheal intubation is not successful.

A commercial silicone 7-14-ID endotracheal tube specific for foals may have been safer than connecting two endotracheal tubes, but was not available in our clinic. The need to connect two endotracheal tubes was due to the fact that tubes with a diameter narrow enough to pass through the nasal cavities were too short to reach the trachea. Our solution was to connect the extremities of two tubes, the smaller nested into the larger one, to create a tube with an adequate length and diameter. The connection was accomplished using adhesive tape, which was a limitation. The major concern was that the tubes might detach from one another, leading to entrapment of the distal tube in the trachea. In that case, the distal tube could have been removed by pulling out the balloon tubing connected to the three-way stopcock that was maintained outside the animal’s mouth. However, despite these concerns, detachment did not occur and the connected tubes worked well. A glue could be added to decrease the risk of detachment of the distal tube. However, we chose not to do so to avoid further decrease of safety by adding another material that could detach in small pieces (difficult to remove) from the tube once wet. The cyanoacrylate glue warms up the material that can be slightly melted, which would also further impair safety.

## Concluding Remarks

The anesthetist should anticipate additional stress and possible respiratory distress during induction and recovery in ponies affected by tracheal collapse and therefore provide preventive measures such as the ones described in this case report. In summary, anesthetic management of these cases should focus on the following: (1) preventing stress by providing good sedation in the induction phase; (2) orotracheal or nasotracheal intubation for surgery; and (3) in the recovery phase, adequate sedation and analgesia and nasotracheal intubation for prolonged extubation.

## Ethics Statement

Owner consent was obtained for including the animals in this case report.

## Author Contributions

KI contributed to the data acquisition, analysis, and interpretation, drafted and revised the work, and approved the final version to be published. AS, AG, and CS contributed to the data acquisition, analysis, and interpretation, revised the work and approved the final version to be published. MG and DS contributed to the data analysis and interpretation, and revised and approved the final version to be published.

## Conflict of Interest Statement

The authors declare that the research was conducted in the absence of any commercial or financial relationships that could be construed as a potential conflict of interest. The authors and the institution did not receive any payment or services from a third party for any aspect of the submitted work.
